# Axillary Recurrence After a Negative Sentinel Lymph Node Biopsy (SLNB) for Initial Positive Node Breast Cancer Postneoadjuvant Therapy: Insights From a Systematic Review and Meta‐Analysis

**DOI:** 10.1155/tbj/8396104

**Published:** 2026-02-24

**Authors:** Maha A. Alghamdi, Hemali Deshpande, Walid M. Abd El Maksoud, Fahad S. Al Amri, Mohammed A. Bawahab, Khaled S. Abbas, Abdullah Dalboh, Hassan A. Alzahrani, Marei H. Alshandeer, Ahmad Jebril M. Bosaily, Haytham M. Fayed, Ibrahim A. Alghamdi

**Affiliations:** ^1^ Department of General Surgery, College of Medicine, King Khalid University, Abha, Saudi Arabia, kku.edu.sa; ^2^ Department of Anatomy, College of Medicine, King Khalid University, Abha, Saudi Arabia, kku.edu.sa; ^3^ College of Medicine, King Khalid University, Abha, Saudi Arabia, kku.edu.sa

**Keywords:** axillary recurrence, meta-analysis, postneoadjuvant therapy, sentinel node biopsy

## Abstract

**Background:**

Despite the use of sentinel lymph node biopsy (SLNB) to stage the axilla in clinically node‐negative breast cancer patients’ postneoadjuvant therapy, the incidence and clinical significance of axillary recurrence (AR) after a negative SLNB remain under‐explored in the literature. Understanding these factors is essential to improving patient outcomes and guiding future treatment strategies.

**Methods:**

We conducted a systematic review and meta‐analysis using PubMed, Scopus, ScienceDirect, and Google Scholar. A random effects model was used to calculate pooled incidence of AR, while heterogeneity was assessed using the I^2^ and Q‐statistics. Publication bias was evaluated with a funnel plot and Egger’s test. We performed all analyses using the R meta and metafor packages.

**Results:**

A total of 37 studies were included in the qualitative synthesis and meta‐analysis. The pooled analysis of these studies highlights a remarkably low incidence of AR, with an overall proportion of 1% (95% CI: [0.0%, 1.0%]) under a random‐effects model and no significant heterogeneity (*I* ^2^ = 0%, *p* = 0.95). Egger’s test for publication bias was conducted to assess the presence of funnel plot asymmetry in the meta‐analysis, indicating no significant evidence of publication bias (*z*‐value = 0.6074, *p*‐value = 0.5436).

**Conclusion:**

The meta‐analysis demonstrated a remarkably low incidence of AR after a negative SLNB in initially node‐positive breast cancer patients postneoadjuvant therapy. These findings suggest that such recurrences are rare, supporting the reliability of SLNB in accurately staging the axilla. This highlights its importance in clinical decision‐making for treatment strategies.

## 1. Introduction

Sentinel lymph node biopsy (SLNB) has become the standard of care for axillary staging in breast cancer due to its demonstrated accuracy and safety in numerous large studies [1–3]. The adoption of SLNB has significantly reduced the need for more invasive axillary lymph node dissection (ALND), which is associated with higher morbidity, including lymphedema and impaired arm mobility [3]. Moreover, in patients with negative findings on SLNB, omitting ALND has been shown to substantially lower the risk of arm morbidity without compromising oncological outcomes.

Despite these advancements, long‐term follow‐up data on axillary recurrence (AR) after negative SLNB remain limited. Reported AR rates following negative SLNB range from 0.4% to 0.9%, but these findings are largely based on follow‐up periods of less than 8 years [4–8]. This raises concerns about the potential for late AR, particularly given the increasing use of systemic therapies and their impact on locoregional recurrence patterns. Late axillary events may be especially relevant for patients with estrogen receptor (ER)‐positive breast cancer, where systemic recurrences are known to manifest even years after the initial treatment [9, 10].

Neoadjuvant chemotherapy (NAC) has further revolutionized breast cancer management by effectively eradicating axillary metastases in nearly half of treated patients, enabling a shift from ALND to SLNB in selected cases [11–13]. This de‐escalation of axillary surgery has reduced morbidity while maintaining oncological safety [14]. However, as systemic therapies continue to evolve, the long‐term impact of these treatment strategies on AR remains uncertain. The critical role of axillary nodal status in determining local and systemic treatment underscores the need for robust evidence on recurrence patterns to optimize patient outcomes.

Given the paucity of long‐term data and the evolving treatment landscape, a systematic review and meta‐analysis are warranted to comprehensively evaluate the incidence and clinical significance of AR after a negative SLNB in patients initially presenting with positive axillary nodes and treated with neoadjuvant therapy. This meta‐analysis aims to address the lingering questions about late AR, particularly in the context of modern systemic therapies, and to provide insights that can inform clinical decision‐making and follow‐up strategies.

## 2. Material and Methods

### 2.1. Search Strategy and Databases

A comprehensive search of the literature was conducted using four databases: PubMed, ScienceDirect, Scopus, and Google Scholar. The search strategy incorporated Medical Subject Headings (MeSH) terms and free‐text keywords to maximize the retrieval of relevant studies. Boolean operators (AND, OR, NOT) were used to combine search terms logically. Specific terms related to AR, SLNB, breast cancer, neoadjuvant therapy, and associated outcomes were included. The search was restricted to studies published in English, and no limits were placed on the year of publication to ensure a thorough capture of available evidence. We included quantitative studies such as randomized controlled trials (RCTs), prospective and retrospective cohort studies, and case series, while letters to the editor, commentaries, and qualitative studies were excluded. Studies assessing AR after a negative SLNB were included, and we also included studies on AR after omitting completion ALND. The studies included in this meta‐analysis utilized various techniques for SLNB detection. According to current guidelines, optimal identification of sentinel lymph nodes involves dual methods, including the use of blue dye and radioactive colloid technetium. However, there was variability in the methods employed across the studies. Some studies adhered to the dual‐method approach, while others relied on a single method for sentinel node detection. Additionally, not all studies reported whether positive lymph nodes were clipped prior to neoadjuvant therapy, which could influence the accuracy of SLNB. This heterogeneity in methodology underscores the need for standardized approaches to improve comparability and reliability of results. We used PICO (Population, Exposure/Intervention, Comparison, and Outcome) criteria to search for and identify eligible studies. A detailed table of search terms used in each database is provided in Table 1.

**TABLE 1:** Search strategy and key search terms using PICO criteria.

PICO element	Search term	MeSH term
Population	“Initial positive node breast cancer” OR “axillary lymph node metastasis” OR “breast cancer patients” OR “neoadjuvant therapy” OR “post‐neoadjuvant therapy” OR “sentinel lymph node biopsy (SLNB)” OR “axillary recurrence” OR “breast cancer recurrence”	“Breast Neoplasms” OR “Axillary Lymph Node Metastasis” OR “Neoadjuvant Therapy” OR “Sentinel Lymph Node Biopsy” OR “Lymphatic Metastasis”
Exposure	“Sentinel lymph node biopsy (SLNB)” OR “neoadjuvant chemotherapy” OR “neoadjuvant therapy” OR “surgery” OR “axillary surgery” OR “neoadjuvant therapy (chemotherapy, radiation)”	“Neoadjuvant Therapy” OR “Sentinel Lymph Node Biopsy” OR “Axillary Lymph Node Dissection” OR “Chemotherapy, Adjuvant” OR “Radiotherapy”
Comparison	“Negative SLNB result” OR “axillary recurrence after SLNB” OR “non‐negative SLNB result” OR “axillary lymph node dissection (ALND)” OR “no intervention” OR “no axillary recurrence”	“Lymphatic Metastasis” OR “axillary Lymph Node Dissection” OR “Sentinel Lymph Node Biopsy” OR “Neoplasm Staging”
Outcome	“Axillary recurrence” OR “recurrence rates” OR “clinical significance of recurrence” OR “survival rates” OR “morbidity” OR “disease‐free survival” OR “prognosis” OR “breast cancer prognosis” OR “long‐term outcomes after SLNB” OR “clinical significance post‐SLNB”	“Axillary Recurrence” OR “Survival Rate” OR “Disease‐Free Survival” OR “Neoplasm Recurrence, Local” OR “Prognosis” OR “Breast Cancer, Secondary” OR “Morbidity”

### 2.2. Data Extraction

Data extraction was performed independently by two reviewers using a standardized data extraction form. The information retrieved from each study included the author’s name, year of publication, study design, age of participants, clinical setting, duration of follow‐up, number of events of AR, and sample size. Discrepancies in data extraction were resolved through consensus or consultation with a third reviewer to ensure accuracy and consistency.

### 2.3. Quality or Risk of Bias Assessment

The methodological quality of included studies was assessed separately for observational studies and RCTs using standardized tools. For observational cohort studies, the Newcastle–Ottawa Scale (NOS) was applied, which evaluates three main domains: selection of study groups, comparability of cohorts, and ascertainment of outcomes. Each study was scored across these domains, with a maximum score of 16 points. Studies were categorized as high (12–16 points), moderate (8–11 points), or low (< 8 points) quality based on their total NOS score. Assessment was performed independently by two reviewers, with discrepancies resolved by discussion or consultation with a third reviewer.

For RCTs, methodological quality was evaluated using the Cochrane Risk of Bias 2.0 (RoB 2) tool, which assesses risk of bias across five domains [1]: randomization process [2], deviations from intended interventions [3], missing outcome data [4], measurement of the outcome, and [5] selection of reported results. Each domain was rated as low risk, some concerns, or high risk, leading to an overall risk‐of‐bias judgment for each trial. Independent assessments were performed by two reviewers, and consensus was achieved through discussion. This dual approach allowed for robust evaluation of study quality while accounting for differences in study design, ensuring that both observational and randomized evidence were appropriately appraised.

### 2.4. Statistical Analysis

Statistical analysis was performed using a random‐effects model to account for heterogeneity among studies. The pooled incidence of AR was calculated, and 95% confidence intervals (CIs) were reported to assess precision. Heterogeneity was evaluated using the I‐squared (*I* ^2^) statistic and Cochran’s *Q* test. A forest plot was generated to display the pooled incidence of AR with 95% CIs. Funnel plots and Egger’s regression test were used to assess the risk of publication bias. All statistical analyses were conducted using R (Packages: meta and metafor), and significance was set at a *p*‐value of less than 0.05.

## 3. Study Results

### 3.1. Flow of Studies

**FIGURE 1:**
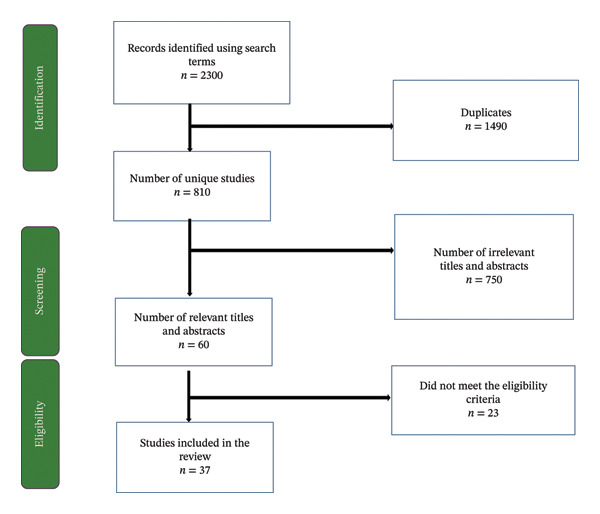
PRISMA flow diagram showing the flow of screening and identification of studies.

A systematic review was conducted to evaluate studies on AR after a negative SLNB in breast cancer patients who underwent neoadjuvant therapy. Initially, a total of 2300 records were identified from databases (PubMed, Scopus, ScienceDirect, Google Scholar). After 1490 duplicates were removed, 810 articles remained for title and abstract screening, during which 750 articles were excluded for not meeting the inclusion criteria. Following this, 60 full‐text articles were assessed, and 23 articles were excluded for reasons such as incorrect study design, irrelevant outcomes, or population. Ultimately, 37 studies were included for qualitative synthesis and meta‐analysis (Figure 1).

### 3.2. Characteristics of Studies Included in Review

Table 2 illustrates the characteristics of the studies included in the review and meta‐analysis. The review and meta‐analysis included a total of 37 studies, providing a comprehensive evaluation of the incidence and clinical significance of AR. Regarding the timeline of publication, 27 studies (approximately 73%) were conducted before 2010, while 10 studies (27%) were conducted during or after 2010. This distribution highlights a consistent interest in the topic over time, with a notable increase in larger studies in recent years. The studies published before 2010 mostly focused on AR after omitting completion ALND, whereas studies published after 2010 assessed AR after a negative SLNB for initial positive node breast cancer postneoadjuvant therapy.

**TABLE 2:** Characteristics of studies included in review (n = 37).

Author	Year	Country	Study design	Event	n	Incidence	Age	Follow up	Subgroup
Liang et al. [15]	2001	United States	Retrospective chart review	0	4	0	55	14 months	Before 2010
Guenther et al. [16]	2003	United States	Prospective cohort study	0	39	0	62	32 months	Before 2010
Ganaraj et al. [17]	2003	United States	Prospective study	0	17	0	53	30 months	Before 2010
Fant et al. [18]	2003	United States	Retrospective chart review	0	27	0	NR	30 months	Before 2010
Fournier et al. [19]	2004	United States	Retrospective chart review	0	6	0	53	12 months	Before 2010
Swenson et al. [20]	2005	United States	Prospective study	1	63	0.0159	59	33 months	Before 2010
Schrenk et al. [21]	2005	Austria	Retrospective cohort	0	16	0	59	48 months	Before 2010
Jeruss et al. [22]	2005	United States	Prospective study	0	73	0	58	28 months	Before 2010
Fan et al. [23]	2005	United States	Retrospective study	1	27	0.037	53	31 months	Before 2010
Chagpar et al. [24]	2005	United States	Retrospective study	0	15	0	57	40 months	Before 2010
Carlo et al. [25]	2005	United States	Prospective study	0	20	0	57	60 months	Before 2010
Schulze et al. [26]	2006	Germany	Retrospective cohort	0	5	0	64	49 months	Before 2010
Haid et al. [27]	2006	Austria	Prospective study	0	8	0	59	47 months	Before 2010
Hwang et al. [28]	2007	United States	Retrospective cohort	0	157	0	56	30 months	Before 2010
Zakaria et al. [29]	2008	United States	Retrospective chart review	0	69	0	62	30 months	Before 2010
Cox et al. [30]	2008	United States	Retrospective cohort	0	25	0	59	20 months	Before 2010
Bilimoria et al. [31]	2009	United States	Population‐based registry	3	530	0.0057	58	64 months	Before 2010
Pernas et al. [32]	2009	Spain	Prospective cohort study	0	45	0	55	60 months	Before 2010
Langer et al. [33]	2009	Switzerland	Prospective study	0	27	0	62	77 months	Before 2010
Yi et al. [34]	2010	United States	RCT	2	1767	0.0011	61	50 months	Before 2010
Giuliano et al. [35]	2010	United States	RCT	1	160	0.0063	54	76 months	Before 2010
Takei et al., 2010 [36]	2010	Japan	Retrospective cohort	0	100	0	55	58 months	Before 2010
Cyr et al. [37]	2010	United States	Retrospective study	1	61	0.0164	57	60 months	Before 2010
Yegiyants et al. [38]	2010	United States	Prospective cohort study	1	33	0.0303	57	79 months	Before 2010
Meretoja et al. [39]	2010	Finland	Retrospective study	0	48	0	67	37 months	Before 2010
Degnim et al. [40]	2010	United States	Retrospective chart review	0	50	0	57	38 months	Before 2010
Pugliese et al. [41]	2010	United States	Retrospective review	0	76	0	59	77 months	Before 2010
Andersson et al. [42]	2012	Sweden	Prospective cohort	22	2195	0.01	NR	65 months	After 2010
Boniface et al. [43]	2017	Sweden	Multicenter cohort study	35	2216	0.0158	NR	10 years	After 2010
Piltin et al. [44]	2020	United States	Retrospective analysis	4	602	0.007	51	34 months	After 2010
Kahler‐Ribeiro‐Fontana et al. [45]	2021	Italy	Retrospective study	11	688	0.016	47	9.2 years	After 2010
Barrio et al. [46]	2021	United States	Retrospective study	1	234	0.004	49	40 months	After 2010
Martelli et al., 2022 [47]	2022	Italy	Prospective cohort	0	353	0	47	108 months	After 2010
Tinterri et al. [48]	2023	Italy	Retrospective study	3	160	0.019	50	43 months	After 2010
Tinterri et al. [49]	2024	Italy	Retrospective analysis	1	112	0.01	51	NR	After 2010
Cabiglu et al. [50]	2024	Turkey	Prospective cohort	0	18	0	45	55 months	After 2010
Marinescu et al. [51]	2024	Ireland	Case series	1	79	0.0127	48	4.4 years	After 2010

*Note: n*: total sample size; event: women who developed axillary recurrence.

Abbreviation: RCT = randomized controlled trial.

Geographically, the majority of studies were conducted in the United States, contributing 22 studies (60%) to the analysis. European countries accounted for 7 studies, including significant contributions from Sweden (*n* = 2), Austria (*n* = 2), and Italy (*n* = 4). Other countries, such as Finland, Japan, Turkey, Germany, Spain, Switzerland, and Ireland, provided one study each. This distribution reflects a dominance of research from high‐income countries, particularly the United States and Europe. In terms of the population age group, most studies included adult patients, typically middle‐aged to older women (45–67 years), reflecting the population most affected by the condition under review.

With respect to the study design, there were retrospective cohort studies or analyses (*n* = 13), RCTs (*n* = 2), case series (*n* = 1), medical chart reviews (*n* = 1), population‐based registries (*n* = 1), and prospective cohort studies (*n* = 13). Detailed age‐specific data were not consistently provided across studies, as shown in Table 2. Follow‐up periods varied widely, ranging from 12 months to over 10 years, with longer follow‐up durations being more common in studies conducted after 2010, as shown in Table 1.

### 3.3. Incidence of AR: Pooled Estimate

**FIGURE 2:**
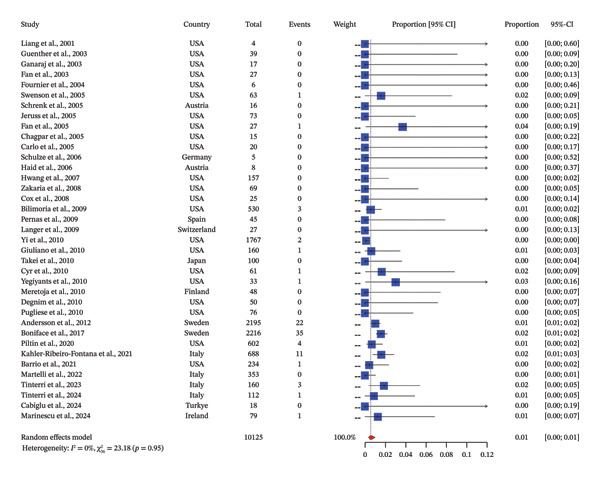
Pooled incidence of axillary recurrence after negative sentinel lymph node biopsy in breast cancer patients postneoadjuvant therapy.

The pooled analysis of 37 studies highlights a remarkably low incidence of AR, with an overall proportion of 1% (95% CI: [0.0%, 1.0%]) under a random‐effects model and no significant heterogeneity (*I* ^2^ = 0%, *p* = 0.95). Studies conducted before 2010 predominantly report no recurrence events [15–33], particularly those with smaller sample sizes, such as Liang et al. [15], Guenther et al. [16], and Swenson et al. [20], reflecting early successes in SLNB and adjuvant therapy. Notable exceptions, such as Chapgar et al. [24] and Yi et al. [34], reported recurrence rates of around 1%–2%, demonstrating the variability linked to larger cohorts. Post‐2010 [34–51], similar trends are observed, with studies like Andersson et al. [42] and de Boniface et al. (2017) [52] significantly contributing to the pooled estimate. With sample sizes exceeding 2000 patients, they reported 22 and 35 recurrence events, respectively, emphasizing the importance of large‐scale data in achieving precise estimates. Collectively, these studies anchor the pooled estimate, while others with zero events across smaller cohorts offer additional support for the rarity of AR (Figure 2).

### 3.4. Publication Bias Findings

#### 3.4.1. Funnel Plot

**FIGURE 3:**
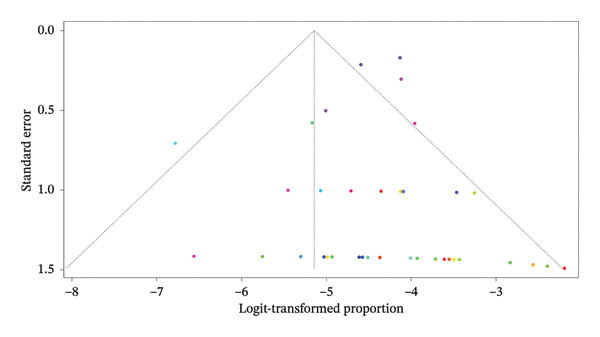
Funnel plot assessing publication bias for the meta‐analysis of axillary recurrence.

The funnel plot provides a graphical assessment of publication bias and the heterogeneity of effect sizes in studies analyzing AR (Figure 3). In this plot, the *x*‐axis represents the logit‐transformed proportion of AR, and the *y*‐axis denotes the standard error. Studies with larger sample sizes (and thus smaller standard errors) appear near the top of the plot, while smaller studies with higher standard errors are plotted toward the bottom. The plot appears relatively symmetric, suggesting that there is no strong evidence of publication bias. Both sides of the pooled estimate line (vertical dashed line) are populated, particularly among studies with larger standard errors, which would typically be more susceptible to bias. Studies are tightly clustered near the bottom‐left of the funnel (with low proportions and small standard errors), reflecting the low incidence of AR. This clustering aligns with the pooled estimate of 1% incidence. A few studies at the bottom‐right and bottom‐left of the plot deviate slightly from the pooled estimate, likely reflecting variability due to small sample sizes rather than systematic bias. In conclusion, the funnel plot does not indicate significant asymmetry, and thus there is no strong evidence of publication bias in this analysis. The low heterogeneity observed in the pooled analysis (*I* ^2^ = 0%) further supports this interpretation. The results appear robust and consistent, with minimal influence from small‐study effects or selective reporting.

#### 3.4.2. Egger’s Regression Test

Egger’s test for publication bias was conducted to assess the presence of funnel plot asymmetry in the meta‐analysis. The results indicated no significant evidence of publication bias, as the *z*‐value was 0.6074 with a *p*‐value of 0.5436. This *p*‐value is well above the commonly used threshold of 0.05, suggesting that the distribution of studies in the funnel plot does not exhibit asymmetry. Additionally, the limit estimate for the regression slope as the standard error approaches zero was −4.5641, with a CI ranging from −5.1088 to −4.0194. Although the CI does not include zero, the lack of statistical significance in the Egger’s test implies that there is no strong indication of publication bias in the included studies. Overall, these results support the robustness of the findings in the meta‐analysis, with no substantial bias affecting the conclusions.

#### 3.4.3. Quality Assessment of Observational Studies

Overall, as shown in Table 3, the observational studies demonstrated moderate quality, with strengths primarily in patient selection and outcome ascertainment. Most studies clearly defined the study population, including patients undergoing SLNB with or without completion ALND, and applied standardized methods for outcome measurement, such as documented AR and follow‐up duration. However, comparability across cohorts was generally limited, as most studies did not adjust for potential confounders or match patients based on key clinical characteristics. The NOS scores ranged from 10 to 11 out of 16, supporting the reliability of findings while highlighting the need for cautious interpretation due to potential residual confounding.

**TABLE 3:** Quality assessment of observational studies.

Study	Year	Country	Selection [8]	Comparability [2]	Outcome [6]	Total [16]	Overall quality
Liang et al.	2001	United States	6	0	4	10	Moderate
Guenther et al.	2003	United States	6	0	4	10	Moderate
Ganaraj et al.	2003	United States	6	0	4	10	Moderate
Fant et al.	2003	United States	6	0	4	10	Moderate
Fournier et al.	2004	United States	6	0	4	10	Moderate
Swenson et al.	2005	United States	6	0	4	10	Moderate
Schrenk et al.	2005	Austria	6	0	4	10	Moderate
Jeruss et al.	2005	United States	6	0	4	10	Moderate
Fan et al.	2005	Taiwan	6	0	4	10	Moderate
Chagpar et al.	2005	United States	6	0	4	10	Moderate
Carlo et al.	2005	United States	6	0	4	10	Moderate
Schulze et al.	2005	Germany	6	0	4	10	Moderate
Haid et al.	2006	Austria	6	0	4	10	Moderate
Hwang et al.	2006	United States	6	0	4	10	Moderate
Zakaria et al.	2007	United States	6	0	4	10	Moderate
Cox et al.	2008	United States	6	0	4	10	Moderate
Bilimoria et al.	2009	United States	6	0	4	10	Moderate
Pernas et al.	2010	Spain	6	0	4	10	Moderate
Langer et al.	2010	Switzerland	6	0	4	10	Moderate
Takei et al.	2010	Japan	6	0	4	10	Moderate
Cyr et al.	2010	United States	6	0	4	10	Moderate
Yegiyants et al.	2010	United States	6	0	4	10	Moderate
Meretoja et al.	2010	Finland	6	0	4	10	Moderate
Degnim et al.	2011	United States	6	0	4	10	Moderate
Pugliese et al.	2011	United States	6	0	4	10	Moderate
Andersson et al.	2012	Sweden	6	0	4	10	Moderate
Boniface et al.	2017	Sweden	6	0	4	10	Moderate
Piltin et al.	2020	United States	6	1	4	11	Moderate
Kahler‐Ribeiro‐Fontana et al.	2021	Italy	6	0	4	10	Moderate
Barrio et al.	2021	United States	6	0	4	10	Moderate
Martelli et al.	2022	Italy	6	0	4	10	Moderate
Tinterri et al.	2022	Italy	6	0	4	10	Moderate
Cabıoğlu et al.	2024	Turkey	6	0	4	10	Moderate
Marinescu et al.	2024	United Kingdom/Ireland	6	0	4	10	Moderate

#### 3.4.4. Quality Assessment of RCTs: Risk of Bias Assessment

As illustrated in Table 4, two randomized studies were evaluated for methodological quality using the Cochrane Risk of Bias 2.0 tool. The ACOSOG Z0011 trial (Giuliano et al., [10]) was a high‐quality RCT with low risk of bias across all domains, including randomization, adherence to interventions, outcome measurement, and selective reporting. In contrast, Yi et al. [34], although frequently cited alongside RCTs, was a registry‐based observational study from the SEER database rather than a true randomized trial. Consequently, it demonstrated a high overall risk of bias, primarily due to the absence of randomization and potential confounding, with some concerns regarding outcome reporting. These assessments underscore that ACOSOG Z0011 provides robust evidence on axillary management, whereas findings from Yi et al. should be interpreted with caution.

**TABLE 4:** Quality assessment (risk of bias) of RCTs.

Study	Randomization process	Deviations from intended interventions	Missing outcome data	Measurement of outcome	Selection of reported results	Overall risk of bias
Yi et al., 2010	High	Some concerns	Low	Low	Some concerns	High
Giuliano et al., 2010	Low	Low	Low	Low	Low	Low

## 4. Discussion

We undertook the current meta‐analysis to synthesize and summarize evidence from published studies on AR following a negative SLNB for initially positive node breast cancer treated with neoadjuvant therapy. Additionally, we also comprehensively evaluated the studies on AR after omitting completion ALND. The evolution of axillary management strategies in breast cancer reflects advancements in both surgical techniques and systemic therapies. This progression highlights the impact of NAC in achieving nodal downstaging and minimizing the extent of axillary surgery [11]. The present analysis builds on these developments, providing a comprehensive evaluation of recurrence patterns and their clinical implications.

Our findings indicate that the incidence of AR after negative SLNB remains low (about 1%), even among patients initially presenting with positive axillary nodes who received neoadjuvant therapy. Our findings are comparable across the settings, as most of the prior studies have reported AR between 0.2% and 0.9% for micrometastatic disease of the sentinel lymph node and approximately 1% for macrostatic disease of the sentinel lymph node [33, 35, 53]. This consistent decline in recurrence rates reflects advancements in systemic therapies, radiotherapy, and surgical precision, affirming the effectiveness of modern treatment strategies.

These findings align with results from older studies where all patients received ALNDs [54]. Patients with positive axillary lymph nodes typically undergo adjuvant systemic therapy, such as hormone therapy, chemotherapy, or both. While this may contribute to the low recurrence rate in the axilla, radiotherapy to the breast during breast‐conserving treatment is also considered a significant factor for the low AR rate. The findings strongly support de‐escalation of axillary interventions in carefully selected patients, given the overall low recurrence risk. Recently, multiple studies have observed that patients with a positive SLNB who primarily underwent breast‐conserving treatment exhibited a low AR rate without the need for completion ALND [16, 28, 55, 56]. The findings also imply that SLNB is a reliable and safe alternative to ALND in carefully selected patients, minimizing surgical morbidity without compromising oncological outcomes. The observed outcomes support the paradigm shift toward de‐escalation of axillary surgery from ALND to SLNB, facilitated by the downstaging of axillary disease achieved through neoadjuvant therapy. This approach minimizes surgical morbidity while maintaining oncological safety.

Additionally, the impact of systemic therapies, including advancements in chemotherapy and targeted treatments, appears to play a significant role in reducing locoregional recurrence [28]. The efficacy of these therapies in eradicating microscopic disease likely contributes to the low observed recurrence rates. Furthermore, extended follow‐ups in these studies improve the reliability of long‐term outcome assessments. This variability in follow‐up durations underscores the importance of time in evaluating outcomes effectively in the included studies. Additionally, variability in follow‐up duration and study designs among the included studies highlights the importance of standardizing methodologies in future research to better quantify long‐term risks and optimize patient care.

### 4.1. Strengths and Limitations

This systematic review and meta‐analysis provide a comprehensive synthesis of the current evidence regarding AR after negative SLNB for initially positive node breast cancer treated with neoadjuvant therapy. A major strength of this study is its focus on studies published after 2010, which align with contemporary clinical practices and incorporate the impact of modern systemic therapies. Additionally, a large number of studies, mainly focusing on AR in the absence of complete ALND, provided useful insights into the literature on AR prior to the era of modern treatment options. Furthermore, the inclusion of a large cohort of patients with varying follow‐up across multiple studies enhances the generalizability of the findings. A larger number of studies have provided sufficient sample sizes to yield reliable data on AR rates. However, it is noteworthy that the majority of these studies have been conducted in developed countries, where healthcare systems and resources are significantly more advanced. This creates a research gap regarding the applicability of these findings to low‐ and middle‐income countries (LMICs), where healthcare infrastructures and access to treatment can differ greatly. To ensure that global health policies and interventions are effective and equitable, there is a pressing need for more studies focusing on populations in LMICs. Such research could provide valuable insights into the challenges and outcomes specific to these regions, thereby enhancing the generalizability and impact of the findings on a global scale.

However, several limitations must be acknowledged. First, the variability in follow‐up durations across the included studies may affect the ability to capture late ARs. Second, differences in study designs, definitions of recurrence, and systemic therapy regimens introduce heterogeneity that may influence the pooled estimates. Third, data on specific patient subgroups, such as those with residual nodal disease post‐NAC, remain limited, warranting further exploration in future studies. Lastly, most included studies were retrospective, reflecting the practical challenges of conducting prospective research or randomized trials for such a rare outcome with very low event rates; hence, the available evidence carries risks of bias, limits the ability to establish causality, and should be interpreted with appropriate caution.

## 5. Conclusion and Future Directions

This meta‐analysis highlights the low incidence of AR after negative SLNB in patients initially presenting with positive axillary nodes treated with neoadjuvant therapy, reinforcing the safety and reliability of this approach. The findings support the de‐escalation of axillary surgery in selected patients, minimizing morbidity without compromising oncological outcomes. However, the potential for late recurrences, particularly in ER‐positive disease, underscores the necessity for extended follow‐up and standardized surveillance protocols.

While this analysis provides valuable insights into AR rates, the heterogeneity in SLNB detection techniques highlights an important gap in the literature. Specifically, the comparison between single‐technique (e.g., blue dye alone or radioactive colloid technetium alone) and dual‐technique detection methods warrants further investigation. Future studies should focus on directly comparing these approaches to determine whether the dual‐method strategy offers superior accuracy and long‐term outcomes, particularly in patients undergoing neoadjuvant therapy. Future research should focus on addressing the limitations identified in this study. Prospective trials with longer follow‐up durations and standardized methodologies are essential to validate these findings and provide more granular insights into recurrence patterns. Additionally, exploring the role of emerging systemic therapies and their integration with surgical strategies will be critical in optimizing outcomes for patients with breast cancer. Enhanced understanding of patient‐specific factors influencing recurrence risk may also aid in personalizing treatment and follow‐up strategies, ultimately improving patient care.

## Author Contributions

Conceived and designed the analysis: Maha A. Alghamdi, Hemali Deshpande, Walid M. Abd, Fahad S. Al Amri, Mohammed A. Bawahab, Khaled S. Abbas, Abdullah Dalboh, Hassan A. Alzahrani, Marei H. Alshandeer, Ahmad Jebril M. Bosaily, and Haytham M. Fayed. Collected the data: Maha A. Alghamdi, Hemali Deshpande, Walid M. Abd, Fahad S. Al Amri, Mohammed A. Bawahab, Khaled S. Abbas, Abdullah Dalboh, Hassan A. Alzahrani, Marei H. Alshandeer, Ahmad Jebril M. Bosaily, and Haytham M. Fayed. Contributed data or analysis tools: Maha A. Alghamdi, Hemali Deshpande, Walid M. Abd, Fahad S. Al Amri, Mohammed A. Bawahab, Khaled S. Abbas, Abdullah Dalboh, Hassan A. Alzahrani, Marei H. Alshandeer, Ahmad Jebril M. Bosaily, and Haytham M. Fayed. Performed the analysis: Maha A. Alghamdi, Hemali Deshpande, Walid M. Abd, Fahad S. Al Amri, Mohammed A. Bawahab, Khaled S. Abbas, Abdullah Dalboh, Hassan A. Alzahrani, Marei H. Alshandeer, Ahmad Jebril M. Bosaily, and Haytham M. Fayed. Wrote the manuscript: Maha A. Alghamdi, Hemali Deshpande, Walid M. Abd, Fahad S. Al Amri, Mohammed A. Bawahab, Khaled S. Abbas, Abdullah Dalboh, Hassan A. Alzahrani, Marei H. Alshandeer, Ahmad Jebril M. Bosaily, and Haytham M. Fayed.

## Funding

The authors extend their appreciation to the Deanship of Research and Graduate Studies at King Khalid University for funding this study through the Large Research Project under grant number RGP2/533/46.

## Conflicts of Interest

The authors declare no conflicts of interest.

## Data Availability

Data sharing is not applicable to this article as no datasets were generated or analyzed during the current study.
